# The relationship between ambulance team’s professional commitment, occupational anxiety, and resilience levels

**DOI:** 10.1186/s12913-024-11158-x

**Published:** 2024-06-11

**Authors:** İbrahim Uysal, Güneş Korkmaz, Çetin Toraman

**Affiliations:** 1 Medical Education, Çanakkale Onsekiz Mart University, Çanakkale, Turkey; 2 Faculty of Medicine, Department of Medical Education, Istanbul Medeniyet University, İstanbul, Turkey

**Keywords:** Emergency ambulance team, Professional commitment, Occupational anxiety, Resilience, Classical test theory, Item response theory, Scale development

## Abstract

**Background:**

Individuals who have the ability to bounce back from stressful events, to recover from their troubles and adverse environmental conditions by getting stronger each time are known as resilient people. Some professions may cause more occupational anxiety than others due to their characteristics and working conditions. In this research, we aimed to develop a professional commitment scale for the ambulance team. Another aim was to analyze the relationships between professional commitment, occupational anxiety, resilience, gender, job, seniority and working unit variables.

**Methods:**

In the study, data were collected from a total of 1142 emergency ambulance workers working in Emergency Ambulance and Emergency Call Centers in 34 different cities in Turkey. Data were collected using the “Professional Commitment of Ambulance Team Scale (PCATS), Occupational Anxiety Scale for Emergency Medical Service Professionals (OASEMSP), and Resilience Scale for Adults (RSA). Scale development analyses were carried out using Classical Test Theory (CTT) and Item Response Theory (IRT). Regression analysis were used to examine the relationships between professional commitment, occupational anxiety, resilience, gender, job, seniority and working unit.

**Results:**

As a result of the exploratory factor analysis (EFA), it was determined that 8 items remaining in the professional commitment scale formed a single-factor structure, explaining 46% of the variance of professional commitment of the team. The Cronbach’s Alpha reliability value was 0.867. Confirmatory factor analysis (CFA) confirmed the results of exploratory factor analysis. The Cronbach’s Alpha reliability coefficient obtained through CTT was 0.868, and the marginal reliability coefficient within the scope of IRT was 0.877. The test-retest reliability coefficient was calculated as 0.832, which indicates that the scale is valid and reliable.

**Conclusions:**

The study revealed that resilience has a positive effect for professional commitment while occupational anxiety has a negative effect for professional commitment. In addition, having a moderate seniority has a negative (reducing) effect for professional commitment. Other variables (gender, job, and working unit) was found to have no significant impact on professional commitment.

**Supplementary Information:**

The online version contains supplementary material available at 10.1186/s12913-024-11158-x.

## Background

Health problems, accidents, fires, earthquakes, terrorist attacks and many similar events that threaten human life are accepted as emergency situations. Life-threatening emergencies have been a part of human life since their existence [1, 2]. One of the first structures established to intervene in such emergencies in the history was St. John Ambulance Association, which is a volunteer powered, charitable organization dedicated to the teaching of first aid [3]. Then, the emergency personnel were trained as first responders in emergencies, and the concept “Emergency Management System (EMS)” came into existence. The earliest EMS is reported to be the rescue team formed after the disastrous fire at the Vienna Ring Theater in 1881 [4]. This has become a model for similar communities around the world. With knowledge and experience from war surgery and traumatology [5], EMS has developed significantly and become a more complex organization [6, 7].

Despite the rapid technological advancements in emergency medical equipment, the most crucial component of a modern emergency system remains the presence of trained human resources to provide the necessary emergency medical services in response to life-threatening health crises individuals encounter. Emergency healthcare professionals, who provide 24/7 service, consist of a medical doctor, first and emergency technician (Paramedic), emergency medical technicians (EMTs), nurse/medical officer and ambulance driver. The organizational structure aims at continuous development to reach the emergency call earlier and to provide more effective health care services through call centers and pre-hospital emergency medical service centers. Although the effort to reach the sick and injured person’s place has become an indicator of the good functioning of the health system, the increasing number of cases, environmental stress factors, violent incidents and critical decisions made in such complex cases have placed great demands on the healthcare professionals.

Studies show that EMS personnel are at risk. Hogya and Ellis [8], examining 254 injury reports reported by EMS personnel, reported an occupational injury rate of 50% for males and 86% for females. Benson and Jacobs [9], in their survey of 439 EMS workers in the UK, reported a 25.4% yearly back injury rate, 20.3% assault rate, and 9.9% yearly trauma injury rate. In the studies conducted with EMS employees, the frequency of traffic accidents, hand injuries, back injuries, violence, infectious diseases and stress cases are common. While in the study conducted by Maguire et al. [10], the occupational mortality rate was reported as 12.7 per 100,000 employees per year for six years. For example, Maguire et al. [11] found that the injury rate for EMS personnel was found to be 34.6 per 100 employees per year, and they stated that this rate was more than twice the national average in occupational accidents. In addition, it is known that traffic accidents pose a risk of serious injury and death. Considering that EMS professionals spend a significant part of their working hours in traffic with insufficient regulations and under time pressure, it is clear that the risk of traffic accidents is much higher.

In general, excessive workload and job dissatisfaction of healthcare professionals are seen as one of the reasons for burnout [12]. Beldon and Garside [13] stated in their study that 94% of ambulance personnel experienced a sense of personal accomplishment within their professional roles, but more than 50% experienced varying levels of burnout and 87% showed moderate or high levels of depersonalization towards their jobs. Beldon and Garside [13] also found that the complex causes of stress are lack of management support, public abuse of ambulance service by people, involuntary work overtime and a poor work-life balance.

Factors such as a demanding work pace with prolonged and uninterrupted shifts, non-ergonomic work environments, and environmental stress factors at the scene, where healthcare providers delivering pre-hospital emergency services must make critical decisions concerning human life in complex cases, constitute the challenging aspects of their profession. In this regard, Professional Commitment, Occupational Anxiety, and Resilience emerge as significant constructs for healthcare providers delivering pre-hospital emergency services, defining crucial aspects of their profession.

### Professional commitment

Blau [14] defines professional commitment as an “attitude” towards the profession, and Meyer, Allen and Smith [15] explained the psychological relationship between the profession and the individual, as well as emotional reactions to the profession. Aranya, Pollock and Amernic [16] used the term “professional commitment” in relation to “dedication to the job one is performing”. Lanchman and Aranya [17] state that professional commitment is related to highlighting the identity, striving for the profession, commitment to professional goals, values, norms and ethical principles. In general, professional commitment can be defined as working voluntarily, making self-sacrifice, focusing on the work and giving oneself, the desire to act in accordance with the aims and values of the profession, and fulfilling professional roles effectively [18–20]. This is why commitment is associated with effort and zeal.

Meyer, Allen and Smith [15] identified three distinct themes of professional commitment: [1] affective commitment [2], continuance commitment and [3] normative commitment. Affective commitment is identified with an emotional desire to stay in the profession. An individual who is affectively devoted to his profession will have the chance to progress and improve himself in his profession. Continuance commitment is synonymous with staying in the profession. An individual with high professional dedication will prefer to stay in the profession without reckoning in the process between leaving the profession and staying. Normative commitment refers to the situation in which the rules of the profession have become a part of the individual’s own personality. In general, if the dedication to the profession is high, the probability of displaying the skills of the professional at the highest level, making efforts to improve his career, and continuing the profession increases [15, 21].

### Occupational anxiety

Health professionals must work in difficult and stressful conditions in the ambulance due to the dynamic structure of the setting, racing against time, intense workload, and providing services for health problems that unexpectedly arise in a limited time period [22]. Therefore, by its very nature, these professionals face a high degree of stress in their working environments every day. Such working environments and adverse conditions may negatively affect healthcare workers, particularly their mental health, and they may encounter emotional, cognitive, physical, and social problems. Therefore, the continuous stress they have every day may turn into mental disorders such as anxiety [23]. In this context, anxiety is a reflection of stressful work [24]. For example, American Psychological Association [25] states that there’s a fine line between stress and anxiety. Although both are emotional responses, stress is typically caused by an external trigger which may be short-term, such as a work deadline or a fight, such as being unable to work, discrimination, or chronic illness. People under stress experience mental and physical symptoms, such as irritability, anger, fatigue, muscle pain, digestive troubles, and difficulty sleeping. However, anxiety is defined by persistent, excessive worries that don’t go away even in the absence of a stressor. In this sense, anxiety is a bigger problem compared to stress.

Akman, Cetin and Toraman [26] defines anxiety as an emotional state in which a feeling of weakness is experienced during preparation for perceived risk and fear. Similarly, Durdukoca and Atalay [27] states that anxiety is a critical state of feelings that cause negative emotions such as timidity, lack of self-confidence and unhappiness, and these feelings may result in many problems on job performance, and it also affects motivation at work in a negative way. In this context, occupational anxiety can be described as a condition that is related to general anxiety but occurs more specifically in the workplace [28]. As all healthcare professions deal with human life and well-being in medical emergencies, occupational anxiety is of great importance to examine through research.

### Resilience

Although there is no universal definition of resilience [29], it can be described as one’s ability or personal qualities to thrive in the face of adversity and recover from setbacks [30, 31]. In occupational context, this concept is defined as the ability to maintain personal and professional well-being to cope with stress and adversity at work [32]. Therefore, we can state that resilience is an ever-changing phenomenon [33] which has both personal and professional context. However, one cannot always be resilient in every difficult situation. For example, while an individual could show great personal strength, courage, and adaptability in a difficult working environment, he may struggle and have tough hurdles to overcome in his personal life [29].

Atkinson, Martin and Rankin [34] state that resilience is very important to healthcare professionals in particular as this field has an important role in both coping with and recovery from significant health problems. In addition, Brown et al. [35] suggest resilience enables healthcare workers to ameliorate stress, reduce their risk of burnout, and maintain wellbeing during difficult times. Therefore, understanding resilience in health professionals is important. Brown et al. [35] state that resilience of healthcare workers is shaped by personal, interpersonal, and work-related factors, and healthcare professionals should have strong social support as well as organizational support in the work environment.

### Professional commitment

Professional Commitment: It is the relative strength of an individual’s commitment to the relevant profession. It denotes the individual’s perspective on the profession and the motivation they possess to remain in their job; this, in turn, reflects the person’s loyalty to the profession and willingness to maintain and sustain its values and objectives. In the literature, although there are scales developed to analyze medical students’ professional commitment [36], nursing professionals’ professional commitment [37], and health science students’ professional identities [38], there are not any data collection tools specifically developed to analyze emergency ambulance team’s risks, stress, their burnout, professional commitment levels, and their motivation sources. Therefore, development of new data collection tools is needed to carry out research on this issue.

### Purpose of study

This study aims to determine the validity and reliability level (psychometrics features) of the “Professional Commitment of Ambulance Team Scale (PCATS)” through CTT and IRT and to examine the relationship between ambulance teams’ professional commitment, occupational anxiety, and resilience levels. Following are the research questions:

1. What is the validity and reliability level of the “Professional Commitment of Ambulance Team Scale (PCATS)” based on the Classical Test Theory (CTT) and Item Response Theory (IRT)?
2. Are occupational anxiety, resilience, gender, job, seniority and working unit variables meaningful explanatory variables on the ambulance teams’ professional commitment level?

## Methods

### Data collection

#### Professional commitment of ambulance team scale (PCATS)

The first data collection tool used within the scope of the research is the “Professional Commitment of Ambulance Team Scale (PCATS)” which was developed by the researchers for this study (*Supplementary File 1*). The following procedure was followed in the development of the scale:

- Within the scope of the research, 30 emergency ambulance personnel were asked to write an opinion essay on “the meaning of professional commitment, and what feelings and thoughts they have to be a member of this profession?“.
- The essays were reviewed and the items that can be used for the data collection tool were listed.
- The first draft set of question items was submitted to the control of two different language experts. This draft was also submitted to four first and emergency aid specialists who are academics in first and emergency programs and who have practiced this profession in the past, and a total of five experts, one of whom is an academician in the field of measurement and evaluation.
- In line with the opinions and suggestions received from the experts, the items in the tool were revised and the final form was generated before the pilot testing.
- There were 25 items in the first draft of the scale. Responses to these items were structured in a five-point Likert type scale format (“strongly disagree”, “disagree”, “partly agree”, “agree” and “strongly agree”).

#### Occupational anxiety scale for emergency medical service professionals (OASEMSP)

Another data collection tool used in this research is the “Occupational Anxiety Scale for Emergency Healthcare Professionals” developed by Sevinç Postacı et al. [22] to examine the professional concerns of emergency medicine specialists. The scale has a five-point Likert-type format (strongly disagree, 1; disagree, 2; partially agree, 3; agree, 4; completely agree, 5) consisting of 22 items, two sub-factors. One of the two sub-factors of the scale is “bodily, physical and vital concerns (BPVC)”. There are 12 items in this sub-factor and the lowest score is 12 and the highest score is 60. The other sub-factor is “concerns about the environment, employees, equipment and environmental factors” (CRSSEEF). There are 10 items in this sub-factor, and the lowest possible score is 10 and the highest score is 50. The Cronbach’s Alpha reliability coefficient is 0.922 for the BPVC factor; It was determined as 0.866 for the CRSSEEF factor and 0.914 for the whole scale. In addition, the confirmatory factor analysis (CFA) revealed that fit indices of this structure of the scale were X2/sd = 3.132; GFI = 0.862; AGFI = 0.803; NFI = 0.851; IFI = 0.899; CFI = 0.892 and RMSEA = 0.077.

#### Resilience scale for adults-turkish version (RSA-TV)

The third data collection used in this study is “Resilience scale for adults (RSA)” which was developed by Friborg et al. [39]. Basım and Çetin [40] carried out the adaptation of the scale to Turkish culture. The scale aims to determine the resilience level of adults. The scale was adapted into Turkish through a sample of 350 students and 262 employees. The Turkish application of the scale was confirmed with six factors, which overlaps with the original scale. These factors are [1] perceptions of self [2], social competence [3], structural style [4], family cohesion [5], perceptions of future and [6] social resources. The fit indices as a result of confirmatory factor analysis revealed that the structure was confirmed (χ2 = 1104, df = 480, χ2/df = 2.3; RMSEA = 0.055; TLI = 0.90; CFI = 0.91). It was determined that the internal consistency coefficients of the factors ranged between 0.66 and 0.81, and the test-retest reliability ranged between 0.68 and 0.81.

### Data analysis

To analyze data, first, it was examined whether there was missing value in the whole data set. No missing data was found. In the first application, the suitability of the data set obtained from 353 people was tested with Kaiser-Meyer-Olkin (KMO) and Bartlett’s Test of Sphericity to determine whether it is possible to apply factor analysis. For the suitability of the data set, a value above 0.800 and a “p” value significance were sought for Bartlett’s [41, 42]. Principal axis factoring (PAF) method is recommended as a factor determination method for the scales in the development stage [43]. Therefore, in this study, the factor was determined by the PAF method.

According to the literature, in fit indices determined for DFA, 0.050 < RMSEA < 0.080 is acceptable for RMSEA, 0 < RMSEA < 0.050 excellent, 0.950 and above perfect for CFI and TLI, 2 < X2/sd for X2/sd < 5 is acceptable, and 0 < X2/sd < 2 is considered the perfect range [42, 44].

It is necessary to examine the assumptions of unidimensionality and local independence in the validity and reliability examinations with Item Response Theory (IRT) for Likert-type scale items [45]. Unidimensionality requires that individuals have a feature (the related items of the assessment tool are for only one feature) that affect the performance of individuals in the assessment tool [46]. Unidimensionality is evaluated with item correlation matrix or EFA and CFA. The local independence assumption was tested using the Q3 statistic [47] and IRT calibrations were made with the “Mirt v.1.30” [48] package included in the R v.4.0.5 program.

In line with the purpose of the research, the relationships between the professional commitment, occupational anxiety, and resilience levels of the ambulance team were tested by regression analysis. In regression analysis, occupational anxiety on professional commitment, resilience, gender (female and male), job (paramedic, emergency medical technician and ambulance driver, medical officer nurse), seniority (0–1 years, 2–5 years, 6–10 years, Explanation levels of 11 years and above) and working unit (emergency operation center, ambulance station and both of them) variables were examined. Multiple linear regression models are used to examine the effect of multiple independent (explanatory, predictive) variables on a dependent (outcome, output) variable [49]. The Professional commitment modeled in multiple linear regression modeling is a continuous variable. While some of the possible explanatory variables (occupational anxiety and resilience variables) that are thought to explain “professional commitment” are continuous variable, some are categorical) variables (gender, job, seniority and working unit. These categorical variables were included in the regression model as dummy variables. The reason for including categorical variables as dummy/artificial variables in the regression analysis is to prevent autocorrelation between variables [50].

#### Ethics Committee approval

This study was conducted with the approval of Çanakkale Onsekiz Mart University Scientific Research Ethics Committee (Date of Approval: 25.03.2021 /No:06/20).

## Results

### Development of PCATS

In the development of “Professional Commitment of Ambulance Team Scale (PCATS)”, the data set obtained from 353 emergency ambulance team members was used. The KMO value of the data set was 0.910, and the Bartlett’s Test of Sphericity value was 1091.562 (*p* < .05). These values revealed that the data set was suitable for performing EFA. In the principal axis factoring (PAF) method, the factor loading, communalities and item total correlation load values weren’t suitable for Item 1, 2, 3, 5, 6, 8, 9, 10, 11, 16, 17, 18, 19, 20, 21, 23 and 24; therefore, they were removed from the measurement tool. When interpreting factor loads, factor loads between ± 0.30 and ± 0.40 are considered to meet the minimum level for interpretation of the structure. Load values of ± 0.050 or higher are considered significant [51]. Also, it was determined that the remaining 8 items in the measurement tool formed a single factor structure and explained 46% of the variance of Professional commitment levels of emergency ambulance workers, and the Cronbach’s Alpha reliability value was 0.867. Corrected item total correlation, initial, extraction communalities and factor loading values of these 8 items are shown in Table 1.

**Table 1:** PCATS corrected item total correlation, initial, extraction communalities loading and factor loading

Items		Factor Loading	Communalities		Corrected Item Total Correlation
			Initial	Extraction	
i14	In order for my profession to be well understood, I become the defender of my profession on all occasions.	0.793	0.545	0.629	0.726
i13	I seek opportunities to improve myself so that I can do my job perfectly.	0.741	0.503	0.548	0.677
i22	I review and learn about updated emergency aid guidelines as soon as they are published.	0.729	0.476	0.531	0.670
i12	I carry out activities to raise awareness of the society so that the importance of my profession is well understood by the society.	0.702	0.456	0.493	0.644
i15	Being called to an emergency makes me excited.	0.651	0.381	0.424	0.608
i25	I am willing to participate in first aid trainings organized for the community.	0.642	0.384	0.412	0.599
i4	It makes me proud to hear people saying positive things about my profession.	0.585	0.326	0.342	0.541
i7	I will always continue to love my profession under any no circumstances.	0.579	0.323	0.335	0.540

As can be seen in Table 1, the corrected item total correlation values of the remaining 8 items in the measurement tool did not fall below 0.500. The initial and extraction communalities values of the remaining items in the measurement tool did not fall below 0.300. These values obtained in the analysis are the values suggested in the literature. In the interpretation of the factor structure, the minimum factor loading values to be considered should range from ± 0.30 to ± 0.40. A factor loading value of ± 0.50 is considered significant. Values of ± 0.70 and above are regarded as indicative of a well-defined structure (51).

Also, whether the 8-item one-dimensional scale structure obtained from the EFA was confirmed. Before this application, an 8-item scale form was created and the application was started using this form. In the second application, data were obtained from 789 first and emergency aid workers. CFA was applied on this data set. In the CFA performed, the factor loading values of the PCATS items did not fall below 0.610. No modifications were required to the model. The fit-index values of the model were examined, and it was determined as X2/df = 4.821, RMSEA = 0.078, CFI = 0.963, TLI = 0.948. The fit indices were validated according to the reference values suggested in the literature [42, 44].

For PCATS, which passed the EFA, CFA and Cronbach’s Alpha analysis stages, analyzes were carried out in the context of IRT to obtain evidence of IRT validity and reliability. These analyses were carried out with the data obtained from 789 emergency ambulance workers in the second application. One-dimensionality, one of the assumptions required to perform IRT analyses, has been proven within the scope of EFA and CFA. Local independence was tested with the Q3 statistic, and no items that impair local independence were detected. Based on this finding, item calibrations were determined for PCATS items with the Generalized Partial Credit Model (GPCM) within the scope of IRT. S_χ2, (degree of freedom), RMSEA and level of significance statistics of the items according to GPCM were conducted. The “a” (item discrimination) and “b” (item difficulty) parameters and standard errors of the items whose model fit was determined according to the GPCM were estimated separately for each item. The results are given in Table 2.

**Table 2:** Item Parameters, Standard Error Values and RMSEA item compliance levels of PCATS according to GPCM

Items	i4	i7	i12	i13	i14	i15	i22	i25
**a** **(SE)**	1.614 (0.143)	0.984 (0.083)	1.645 (0.133)	2.513 (0.207)	2.105 (0.173)	1.628 (0.135)	1.833 (0.147)	0.931 (0.078)
**b1** **(SE)**	-2.267 (0.418)	-1.498 (0.217)	-2.265 (0.191)	-1.770 (0.113)	-2.405 (0.248)	-2.414 (0.233)	-1.944 (0.164)	-1.879 (0.221)
**b2** **(SE)**	-1.580 (0.175)	-1.768 (0.176)	-1.198 (0.100)	-1.119 (0.072)	-1.828 (0.121)	-1.395 (0.117)	-1.569 (0.106)	-1.454 (0.155)
**b3** **(SE)**	-1.953 (0.145)	-0.696 (0.112)	-0.819 (0.082)	0.300 (0.058)	-0.838 (0.068)	-1.102 (0.091)	-0.252 (0.065)	-0.402 (0.113)
**b4** **(SE)**	-0.338 (0.069)	0.556 (0.105)	0.859 (0.079)		0.377 (0.063)	0.141 (0.070)	1.005 (0.080)	0.659 (0.115)
**S_χ2**	169.232	323.175	139.957	173.408	180.083	194.168	193.134	217.589
**df**	35	51	39	32	29	38	37	52
**RMSEA**	0.070	0.079	0.057	0.075	0.080	0.072	0.073	0.064

SE: Standard Error, S_χ2 : Rest Score or Summed Score, Priors and Violation of Normality, df: Degree of freedom, RMSEA: Root Mean Square Error of Approximation

The cutoff value for RMSEA, which is one of the important fit indices for measurements made with IRT, is 0.08, and six of this value indicates item fit [52]. According to the item fit statistics in Table 2, it was determined that all of the items had RMSEA values of 0.08 and below. Based on this result, it was determined that 8 items of PCATS provided model fit according to GPCM.

**Fig. 1:**
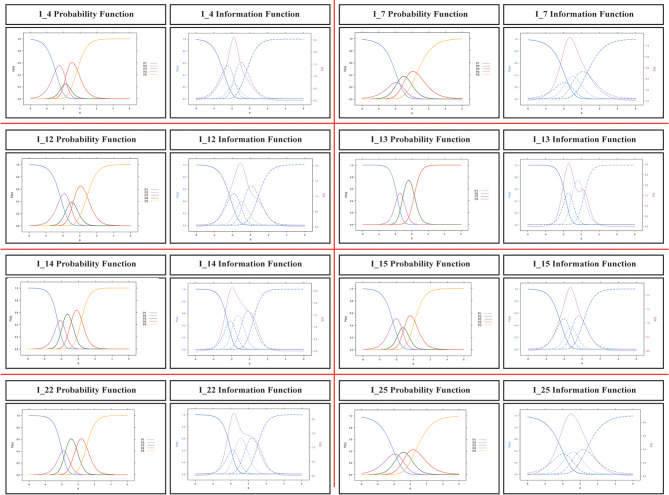
Item characteristic curves and item information functions of PCATS Items

In IRT, the discrimination value of an ideal scale item (parameter “a”) should be between 0.5 and 2. In the literature, the range of this parameter between 0.75 and 2.50 indicates that it is in the acceptable range [53]. Table 2 values showed that the discrimination values of items i4, i7, i12, i14, i15, i22 and i25 were at the desired level. The ideal (medium difficulty level) limits for item difficulty levels (i.e. parameter “b”) are considered to be between − 1.00 and 1.00 [54]. Estimates made according to GPCM (Iteration = 57 LogLikelihood= -6748.934, *p* < .05) prove the consistency of the measurement tool items. Item characteristic curves and information functions are shown in Fig. 1.

According to the item characteristic curves seen in Fig. 1, the items in the measurement tool were functional and distinctive for different levels of the feature of interest. Response categories of the items in the measurement tool were understood by the participants and served as a discriminator. The item information function is a graphical representation that shows the range of the feature (the feature that is tried to be measured in the scale) by which the item best distinguishes the individuals who take the measurement tool [55]. In the item information function, the higher the peak of the curve, the more information the item gives. When PCATS item information functions are examined, items that give less information compared to other items are i7 and i25. Finally, the marginal reliability coefficient of PCATS within the scope of IRT was calculated as 0.877. The marginal reliability coefficient calculated within the scope of IRT was close to the Cronbach’s Alpha coefficient and showed high reliability.

A second application was conducted to perform CFA. In this application, data were obtained from 789 first and emergency aid workers. Out of 789 first and emergency aid workers in this group, 135 people voluntarily provided their contact information. Two months after the second application data were obtained, these 135 first and emergency workers were contacted again and PCATS was applied. In order to determine test-retest reliability with the obtained data, the correlation value between these two applications was calculated. The calculated correlation value was high and significant (*r* = .832, *p* < .05). According to these results, it was interpreted that the test-retest reliability level of PCATS was high.

### The explanatory level of the occupational anxiety, resilience, gender, job, seniority and working unit variables on the professional commitment level of the emergency team

In the second application, PCATS, OASEMSP, RSA-TV scales were applied to 789 emergency ambulance and emergency call center personnel. The relations between professional commitment, occupational anxiety, resilience, gender, job, seniority and working unit with the data obtained as a result of the application were examined with the regression model.

- Response variable (y): Professional commitment (continuous variable).
- Predictor variables (x):

1. Are occupational anxiety, resilience, gender, job, seniority and working unit variables meaningful explanatory variables on the ambulance teams’ professional commitment level?
2. Occupational Anxiety (X1) (continuous variable).
3. Resilience (X2) (continuous variable).
4. Gender (X3): Female and male (categorical variable).
5. Job (X4): Paramedic, emergency medical technician, ambulance driver, medical officer and nurse (categorical variable).
6. Seniority (X5): 0–1 years, 2–5 years, 6–10 years, 11 years and above (categorical variable).
7. Working unit (X6): Emergency operation center, ambulance station and both of them (categorical variable).

Variable 3, 4, 5 and 6 are categorical variables. These variables were included in the regression model as dummy variables. In regression models, when dummy variables are included in the model, one of the levels of the variable is chosen as a reference group, and the analysis results are interpreted accordingly. In the regression model, the reference group of the gender variable is female, the reference group of the job variable is medical officer and nurse, the reference group of the seniority variable is 0–1 year, and finally the reference group of the working unit variable is the emergency operation center group. Regression analysis interpretations were made according to these reference groups. In the regression analysis, the model fit was tested. Model fit results are given in Table 3.

**Table 3:** Model fit for regression model

*R*	*R*²	Model ANOVA				DW Statistic
		F	df1	df2	**p**	
0.930	0.865	310	16	772	< 0.0001	1.87

R = Regression Model Statistic, R^2^ = Regression Model Determination Coefficient, F = Model Fit ANOVA F Test, df = Degree of Freedom, p = Probability of Significance, DW = Durbin Watson Autocorrelation Statistic

Significance (*p* < .05) of the ANOVA test result, which tests the model fit, indicates that the model is suitable. Durbin-Watson (DW) Statistics examines the level of autocorrelation between regression variables. The obtained DW statistics showed that there was no autocorrelation among the variables included in the model. The R2 value indicates the total explanatory level of modeled predictor variables on the professional commitment. In this established regression model, modeled predictor variables explain 86% of the variability on the professional commitment. This high explanatory power suggests that the factors included in our model are significant predictors of professional commitment. However, it is important to note that 14% of the variance remains unexplained. This indicates that there are additional factors influencing professional commitment that were not captured in our model. As the model fit was proven, the interpretation of the regression coefficients of the variables in the model was started. The results are given in Table 4.

**Table 4:** Explanatory variables on professional commitment (Regression Model)

Predictor	β (CI 95%)	t	*p*	VIF
Intercept	8.67 (5.81–11.53)	5.96	< 0.0001	
Social Resources (SR)	0.07 (0.02–0.12)	2.91	0.004	2.46
Family Cohesion (FC)	0.21 (0.16–0.26)	8.88	< 0.0001	2.09
Social Competence (SC)	0.10 (0.02–0.19)	2.40	0.017	2.17
Structured Style (SS)	1.62 (1.47–1.78)	20.17	< 0.0001	2.57
Perception of Future (PF)	0.22 (0.09–0.35)	3.34	< 0.0001	1.75
Perception of Self (PS)	0.19 (0.15–0.24)	8.77	< 0.0001	1.34
Concerns Regarding Setting, Staff, Equipment and Environmental Factors (CRSSEEF)	-0.08 ([-0.11]- [-0.06])	-6.28	< 0.0001	1.97
Bodily, Physical and Vital Concerns (BPVC)	-0.10 ([-0.13]- [-0.08])	-9.61	< 0.0001	1.89
Male	-0.10 ([-0.41]− 0.21)	-0.61	0.540	1.10
Paramedic	0.18 ([-0.42]− 0.78)	0.58	0.562	4.09
Emergency Medical Technician	-0.05 ([-0.65]− 0.54)	-0.17	0.863	4.12
2–5 Years	-0.68 ([-1.24]- [-0.12])	-2.40	0.017	3.21
6–10 Years	-0.95 ([-1.55]- [-0.36])	-3.16	0.002	3.12
11 Years and Above	-0.45 ([-1.04] -0.14)	-1.50	0.135	3.49
Ambulance Station	-0.02 ([-0.56] -0.52)	-0.07	0.946	3.01
Both of Them	-0.23 ([-0.80] -0.35)	-0.77	0.441	2.77

β = Estimate, CI = Confidence Interval, t = T Value of Variable, p = Probability of Significance, VIF = Variance Inflation Factor

The variance inflation factor (VIF) level of each of the variables included in the model was examined. VIF shows multicollinearity between predictive variables. In the event that VIF is “1”, there are not multicollinearity between predictive variables. There is multicollinearity between predictive variables in the event of 1 ≤ VIF ≤ 10. Tolerance is simply the inverse of the Variance Inflation Factor (VIF). Lower tolerance values indicate a higher likelihood of multicollinearity among the variables. A VIF value of 1 suggests that the independent variables are not correlated. If the VIF is between 1 and 5, it indicates moderate correlation among the variables. VIF values between 5 and 10 are problematic, as they signify high correlation among the variables. When the VIF is greater than or equal to 5 and up to 10, multicollinearity is present among the predictors in the regression model. A VIF greater than 10 indicates severe multicollinearity, leading to unreliable estimates of the regression coefficients [56–58]. In the event of VIF > 10, there is a strong multicollinearity between predictive variables. It was determined that the VIF values of the predictor variables included in the regression model do not create multicollinearity.

The intercept is significant in the regression model (*p* < .05). In this case, it means that some variables that are not included in the model have a chance to be a meaningful predictor on the professional commitment.

RSA-TV six sub-factors were included in the regression model. The variables SR, FC, SC, SS, PF and PS are all meaningful (*p* < .05) predictors and explanators of the professional commitment. All sub-factors of Resilience are positive explanators of the professional commitment variable. Structured Style (SS) reached the highest level of explanatory. In this case, it can be said that as resilience increases, the level of professional commitment will also increase.

Two sub-factors of OASEMSP were included in the regression model. The CRSSEEF and BPVC variables are meaningful (*p* < .05) predictors and explanators of the professional commitment. Occupational anxiety is a negative predictor of professional commitment. In this case, it can be said that as the level of occupational anxiety increases, the level of professional commitment will decrease.

Being a male is not a meaningful predictor or explanator of the professional commitment compared to being a female (*p* > .05). Being a paramedic or emergency medical technician is not a meaningful predictor or explanator of the professional commitment when compared to being an ambulance driver, medical officer and nurse (*p* > .05). Having 11 years or more seniority is not a meaningful predictor or explanator of the professional commitment when compared to having 0–1 years seniority (*p* > .05). But having 2–5 years and 6–10 years seniority is a meaningful (*p* < .05) predictor and explanator of the professional commitment when compared to having 0–1 years seniority. It can be said that moderate seniority reduces professional commitment. Having served in the ambulance station or both ambulance station and emergency operation center is not a meaningful predictor and explainer of the professional commitment compared to only having worked in the emergency operation center (*p* > .05).

## Discussion

Despite an extensive review of the literature, the researchers found no studies on the relationship between ambulance teams’ resilience, occupational anxiety, and professional commitment levels. However, there have been found some studies that focus on these variables and their relationships in other health professional’s context. We believe that discussing similar and different findings related to these variables will contribute to the literature as they include participants from health professionals.

Based on the findings from this study, resilience has a positive (increasing) effect on professional commitment. In literature, there are several studies which are in line with this finding. For example, in the study conducted by Gerami Nejad et al. [59] to investigate the relationship between resilience and professional commitment among nurses working in intensive care unit, it was found a positive and significant relationship between resilience and profession commitment. Similarly, Yu et al. [60] found that newly graduated male nurses perceive a moderate degree of resilience, and resilience is positively correlated with professional commitment. In a study conducted during COVID-19 revealed that there was a significant direct relationship between professional commitment and resilience in the nurses working in COVID-19 units [61]. On the other hand, there are some studies the results of which diverge from our study. For example, in the study carried out to test the professional commitment, resilience and intent to leave the profession among nurses during the COVID-19 pandemic, Kleier et al. [62] found resilience was not a predictive of professional commitment.

The findings of this study have also shown that occupational anxiety has a negative (reducing) effect on professional commitment. In other words, if occupational anxiety level increases, professional commitment levels decrease. This finding is in line with the study conducted by Dorevitch and Forst [63] in that stress and anxiety at work may also cause reduced professional commitment in emergency care personnel and negatively influence the quality of care. Similarly, Lambert and Paoline [64] found that those who experience higher levels of stress from the job might be less likely to bond with their profession. Alexander et al. [65] stated that increasing occupational stress and anxiety negatively affects professional and occupational commitment levels of paramedics. In a study conducted with nurses, midwives, and paramedics in Australia by Glass et al. [66], it was also found that workplace overload caused stress and occupational anxiety and this had adverse effect on their commitment levels. On the other hand, having a moderate level of seniority has a negative (reducing) effect on professional commitment, and other variables (gender, job and working unit) have no significant impact on professional commitment. Unfortunately, we couldn’t reach any studies on these variables and professional commitment.

In addition, although the factors included in our model are significant predictors of professional commitment, 14% of the variance remains unexplained. Therefore, we suggest future research should aim to identify and investigate the factors contributing to the remaining 14% of unexplained variance in professional commitment. Potential areas of exploration could include variables not considered in the current study, such as personal values, organizational culture, or external socio-economic factors. Additionally, qualitative studies could provide deeper insights into the complex and nuanced influences on professional commitment that are not easily quantifiable. By exploring these additional factors, future studies can further enhance the understanding and predictive power of models related to professional commitment.

## Conclusions

Measurement tools can be developed according to different theories. Classical test theory (CTT) is a widely used test development theory. Traditionally developed measurement tools are considered in the context of the CTT. The ease and practicality of calculations in the scale development process makes CTT stand out. However, measurement tools developed according to this theory have some limitations. For example, the psychometric properties of a measurement tool developed according to the CTT are dependent on the group to which the tool is applied. In addition, a single standard error value can be obtained for an entire group in measurement tools developed according to CTT. In Item Response Theory (IRT), on the other hand, item parameters are independent of the respondent group, and similarly, group characteristics are independent of the item sample. In addition, separate standard error estimation is possible for each respondent [46].

In the literature, there are professional commitment scales for medical students [36], for nursing professionals [37], and for health students [38]. However, “Professional Commitment of Ambulance Team Scale (PCATS)” developed in this study the first scale specifically designed for ambulance teams. As a result of the explanatory factor analysis in the study, it was determined that the remaining 8 items in the measurement tool formed a single factor structure, explained 46% of the variance of the staff’s dedication to their profession, and the Cronbach’s Alpha reliability value was 0.867. Analyses performed within the scope of Confirmatory Factor Analysis confirmed the results of EFA. The Cronbach’s Alpha reliability coefficient calculated with CTT was 0.868, and the marginal reliability coefficient calculated through IRT was 0.877. The test-retest reliability coefficient was calculated as 0.832. These results showed that the reliability of this scale is high.

In the principal axis factoring (PAF) method, the factor loading, communalities and item total correlation load values weren’t suitable for Item 1, 2, 3, 5, 6, 8, 9, 10, 11, 16, 17, 18, 19, 20, 21, 23 and 24; therefore, they were removed from the measurement tool. These items are as follows: Item 1”Providing emergency assistance is highly sacred”, Item 2 “I take pride in providing emergency assistance”, Item 3 “Negative thoughts about my profession discourage me”, Item 5 “Sometimes I wonder why I chose such a demanding profession”, Item 6 “Regardless of positive or negative thoughts, I am a staunch advocate of my profession”, Item 8 “Despite the challenging behaviors of the people we serve, I continue my profession with dedication”, Item 9 “I feel that my profession is becoming more undervalued day by day”, Item 10 “I have always dreamed of providing emergency assistance throughout my life”, Item 11 “If I could go back in time, I would not choose a profession that provides emergency assistance”, Item 15 “Being called to an emergency case brings me great joy”, Item 16 “Even in cases I don’t consider urgent, I maintain my professional approach as required by my profession”, Item 17 “Emergency assistance is at the center of my life”, Item 18 “My morale is lowered when our team is announced over the radio during a shift”, Item 19 “I feel great satisfaction every time I deliver a case to the hospital”, Item 20 “I feel happy knowing that I did everything I could after intervening in emergency cases”, Item 21 “I feel tired and sad after every case I attend”, Item 23 “I attend in-service training because it is mandatory” and Item 24 “I am excited to share the knowledge and experiences I have learned”. These items were not sufficiently understood by the participants or did not provide enough variance.

Identifying the factors that negatively affect the professional commitment levels of emergency ambulance professionals working under high environmental stress factors throughout their professional life and initiating improvement activities is of great importance for the sustainability of this health service. This service directly affects human life in emergency situations and has a huge impact on mortality and morbidity rates. We suggest the policy makers and hospital managers should develop and apply appropriate strategies to reduce occupational stress, increase their motivation and resilience levels at work. We believe, in this way, they may increase their professional commitment, and thus contribute to their productivity in the workplace.

### Electronic supplementary material

Below is the link to the electronic supplementary material.

Supplementary Material 1

## Data Availability

Available upon request from the corresponding author.

## Abbreviations

PCATS: Professional Commitment of Ambulance Team Scale
OASEMSP: Occupational Anxiety Scale for Emergency Medical Service Professionals
RSA: Resilience Scale for Adults
CTT: Classical Test Theory
IRT: Item Response Theory
CFA: Confirmatory Factor Analysis
EFA: Exploratory factor analysis
