# Supplementary material for: The relationship between ambulance team’s professional commitment, occupational anxiety, and resilience levels
Source: BMC Health Serv Res. 2024 Jun 11;24:716. doi: 10.1186/s12913-024-11158-x (PMC11165765; doi:10.1186/s12913-024-11158-x)
Supplement: Supplementary file 1 — Supplementary Material 1 [file 12913_2024_11158_MOESM1_ESM.docx]

**Supplementary File 1**

**PROFESSIONAL COMMITMENT OF AMBULANCE TEAM SCALE (PCATS)**

Dear Emergency Ambulance Personnel,

We know that you work nonstop to save human lives with great sacrifice every day. Your job is a very difficult one. We are curious about your thoughts on your profession as a member of an emergency ambulance team. This scale is about your thoughts about the profession. What is expected of you is to read each statement and mark the grade that best reflects your opinion for that statement with an “X”. Please do not leave any statements blank. Thank you for your participation.

| **Items** | | **Strongly Disagree (1)** | **Disagree (2)** | **Partly Agree (3)** | **Agree (4)** | **Strongly Agree (5)** |
| --- | --- | --- | --- | --- | --- | --- |
| **1** | In order for my profession to be well understood, I become the defender of my profession on all occasions. | (1) | (2) | (3) | (4) | (5) |
| **2** | I seek opportunities to improve myself so that I can do my job perfectly. | (1) | (2) | (3) | (4) | (5) |
| **3** | I review and learn about updated emergency aid guidelines as soon as they are published. | (1) | (2) | (3) | (4) | (5) |
| **4** | I carry out activities to raise awareness of the society so that the importance of my profession is well understood by the society. | (1) | (2) | (3) | (4) | (5) |
| **5** | Being called to an emergency makes me excited. | (1) | (2) | (3) | (4) | (5) |
| **6** | I am willing to participate in first aid trainings organized for the community. | (1) | (2) | (3) | (4) | (5) |
| **7** | It makes me proud to hear people saying positive things about my profession. | (1) | (2) | (3) | (4) | (5) |
| **8** | I will always continue to love my profession under any no circumstances. | (1) | (2) | (3) | (4) | (5) |
